# Antinociception produced by *Thalassia testudinum *extract BM-21 is mediated by the inhibition of acid sensing ionic channels by the phenolic compound thalassiolin B

**DOI:** 10.1186/1744-8069-7-10

**Published:** 2011-01-24

**Authors:** Anoland Garateix, Emilio Salceda, Roberto Menéndez, Erik L Regalado, Omar López, Teidy García, Ruth A Morales, Abilio Laguna, Olivier P Thomas, Enrique Soto

**Affiliations:** 1 Centro de Bioproductos Marinos, Agencia de Medio Ambiente, Ministerio de Ciencia, Tecnología y Medio Ambiente, Loma y 37, Alturas del Vedado, C.P. 10600, La Habana, Cuba; 2 Instituto de Fisiología, Benemérita Universidad Autónoma de Puebla, 14 sur 6301, CU, San Manuel, Puebla, Pue., CP 72570, México; 3 Université de Nice-Sophia Antipolis, Laboratoire de Chimie des Molécules Bioactives et des Arômes, UMR 6001 CNRS, Institut de Chimie de Nice, Faculté des Science, Parc Valrose, Nice, France

## Abstract

**Background:**

Acid-sensing ion channels (ASICs) have a significant role in the sensation of pain and constitute an important target for the search of new antinociceptive drugs. In this work we studied the antinociceptive properties of the BM-21 extract, obtained from the sea grass *Thalassia testudinum*, in chemical and thermal models of nociception in mice. The action of the BM-21 extract and the major phenolic component isolated from this extract, a sulphated flavone glycoside named thalassiolin B, was studied in the chemical nociception test and in the ASIC currents of the dorsal root ganglion (DRG) neurons obtained from Wistar rats.

**Results:**

Behavioral antinociceptive experiments were made on male OF-1 mice. Single oral administration of BM-21 produced a significant inhibition of chemical nociception caused by acetic acid and formalin (specifically during its second phase), and increased the reaction time in the hot plate test. Thalassiolin B reduced the licking behavior during both the phasic and tonic phases in the formalin test. It was also found that BM-21 and thalassiolin B selectively inhibited the fast desensitizing (τ < 400 ms) ASIC currents in DRG neurons obtained from Wistar rats, with a nonsignificant action on ASIC currents with a slow desensitizing time-course. The action of thalassiolin B shows no pH or voltage dependence nor is it modified by steady-state ASIC desensitization or voltage. The high concentration of thalassiolin B in the extract may account for the antinociceptive action of BM-21.

**Conclusions:**

To our knowledge, this is the first report of an ASIC-current inhibitor derived of a marine-plant extract, and in a phenolic compound. The antinociceptive effects of BM-21 and thalassiolin B may be partially because of this action on the ASICs. That the active components of the extract are able to cross the blood-brain barrier gives them an additional advantage for future uses as tools to study pain mechanisms with a potential therapeutic application.

## Background

Acid-sensing ion channels (ASICs) are a group of sodium-selective ion channels activated by extracellular acidosis. They are widely expressed in neurons of both the central and the peripheral nervous system and they have also been detected in nonneuronal tissues [1-3]. The participation of the ASICs in sensory processes, synaptic plasticity, learning and memory, and in the survival of neurons following global ischemia in the brain has been demonstrated in various animal models [2,4-8]. The role of ASICs in nociception has been shown in different models of pain and inflammatory hyperalgesia [9,10]. The cutaneous pain produced in humans by moderate pH (up to 6) is largely mediated by the ASICs [11,12] The pain sensation produced by cardiac ischemia seems also to be mediated by ASIC activation [13].

The search for new selective pharmacological agents with no significant side-effects is an increasing requirement for the development of new drugs to be used in the treatment of acute and chronic pain. The standardized extract named BM-21 obtained from the sea grass *Thalassia testudinum*, a marine plant highly abundant along the coasts of Cuba, has been characterized and patented [14]. This product is rich in flavonoid compounds and it does not possess any known toxicity, but has antiinflammatory and antioxidant properties and contributes to the recovery of irradiation-damaged dermis and the normal properties of the epidermis [15,16]. The bioassay-guided fractionation of the plant extract resulted in the isolation of thalassiolin B (chrysoeriol 7-β-D-glucopyranosyl-2"-sulphate), the most abundant component of the extract. Its topical application (240 μg cm^-2^) markedly reduced skin UVB-caused damage. Thalassiolin B scavenged the 2,2-diphenyl-2-picrylhydracyl radical (EC50 = 100 μg mL^-1^) suggesting that it may contribute to the protective effect on the skin of the crude extract of *T. testudinum *[17].

In this study we describe the antinociceptive effects of BM-21 and thalassiolin B on different animal models of pain, and the inhibitory action of BM-21 and thalassiolin B on the ASIC currents in isolated dorsal-root ganglion (DRG) neurons in a primary culture.

## Methods

### Sea Grass Collection

*T. testudinum *(Banks ex König, 1805) was collected in March 2008 from La Concha Beach (22°05'45''N, 82°27'15''W) and identified by Dr. J.A. Areces (Institute of Oceanology, Havana, Cuba). A voucher sample (No. IdO 039) has been deposited in the herbarium of the Cuban National Aquarium.

### Extraction and Isolation

Whole dry and ground *T. testudinum *leaves (840 g) were continuously extracted (three times) with EtOH-H2O (50:50, v:v) during 24 h at room temperature. The combined extracts were filtered and concentrated under reduced pressure and a temperature 30 to 40 °C to yield 54 g of crude extract (BM-21). The fractionation of BM-21 was done by a bioassay-guided protocol, in which BM-21 was dissolved in water and successively partitioned with *n*-hexane, chloroform, and water-saturated *n*-butanol. The bioactive *n*-butanol fraction was then fractionated by size-exclusion column chromatography (Sephadex LH-20, EtOH 97%) to give a late-eluting yellow band. This fraction was further purified by semipreparative C18 reversed-phase HPLC (Phenomenex Luna C18 column, 5 μm, 250 × 10 mm) using a linear gradient of H2O:MeOH from 80:20 to 0:100 in 35 min (flow: 3 mL/min) to yield pure thalassiolin B. The structure was confirmed by spectroscopic analyses (1D and 2D NMR experiments) and comparison with the literature data [18].

### Quantification of thalassiolin B in the crude extract

Aqueous solutions of thalassiolin B were prepared at nine concentrations (0.1 to 3.0 mg mL^-1^), and 5 μL of each was injected into the Waters HPLC instrument (Phenomenex Luna C18 column, 5 μm, 150 × 4.60 mm) with a gradient of H2O: methanol:trifluoroacetic acid (MeOH:TFA) (flow 1.0 mL/min from 80:20:0.1 to 0:100:0.1) as the eluent and with the UV detection set at 280 nm. A standard curve relating concentration to peak area was generated, and the retention time and absorbance spectrum for the compound were recorded. Four replicates of the *T. testudinum *water extract were prepared as described. Following centrifugation, drying, and weighing, these extracts were brought up to 13 mg/mL in water and 5 μL of each was injected in the same HPLC conditions as for the standard. Peak areas corresponding to thalassiolin B were recorded and converted to concentration by using the standard curve (*R *= 0.997).

### Instrumental analyses

Column chromatography was done using Sephadex LH-20 (Pharmacia, Sweden). HPLC purification and quantification of thalassiolin B were made on a Waters 600 system equipped with a Waters 717 plus autosampler, a Waters 996 photodiode array detector, and a Sedex 55 evaporative light-scattering detector (Sedere, France). All solvents used for HPLC were Fisher HPLC grade. The NMR spectra were made on a Bruker Avance 500 MHz spectrometer. Chemical shifts (*δ *in ppm) are referenced to the carbon (*δ*C 49.0) and residual proton (*δ*H 3.31) signals of CD3OD. Low resolution electrospray ionization (ESI) mass spectra were obtained with a Bruker Esquire 3000 Plus spectrometer in the negative mode.

### Studies in vivo

#### Animals

Experiments were made on male OF-1 mice (25-30 g) obtained from the National Center for Laboratory Animals (CENPALAB), Santiago de Las Vegas, La Habana, Cuba. The animals were adapted to the laboratory conditions for at least one week, with free access to food and water, with light:dark cycle 12:12 at a constant room temperature of 20°C and 70% relative humidity. They were maintained in the experimental soundless room for at least 1 day before the experiment and with a restriction of food and water 2 h before it. All experiments were made between 0800 and 1300. All procedures were done according to the European Commission guidelines for the use and care of laboratory animals and approved by the Committee for Animal Care in Research of the Center. The minimum number of animals and duration of observation required to obtain consistent data were used.

#### Drugs and extract administration

The quantity of BM-21 to be used was daily dissolved in distilled water. Indomethacin (IMEFA, Havana, Cuba) was dissolved in 10% Tween 20 (Merck, Darmstadt, Germany), formaldehyde (Merck) in PBS (BDH Lab Supplies, UK) and acetic acid (Merck) in water. Amiloride (Sigma-Aldrich, St. Louis MO) and thalassiolin B were dissolved in polyethylene glycol (Sigma-Aldrich) at 1.5%. Different concentrations of BM-21 and indomethacin were administered orally (by intragastric canulla), whereas 0.01 mL g^-1 ^body weight of acetic acid, amiloride, and thalassiolin B were administered intraperitoneally or 20 μL of formaldehyde was administered subcutaneously. Indomethacin, a well-known peripheral antiinflammatory (cyclooxigenase-1 [COX-1] inhibitor) and analgesic drug, was used as a positive control in all the models. The acetic acid and formaldehyde were used to produce different modalities of pain in the respective tests.

To study the antinociceptive activity of BM-21, the following groups and protocols of treatment were used; Group 1 (control), mice were treated with distilled water and served as normal vehicle control. Groups 2, 3, 4, 5 and 6 mice were treated with BM-21 (4, 20, 40, 400, and 1000 mg kg^-1^). Group 7, animals were treated with 15 mg kg^-1 ^indomethacin. Another experimental series included the study of the effects of both 100 mmol kg^-1 ^amiloride and 100 mmol kg^-1 ^thalassiolin B on the formalin test. Each animal was used once. The administration of the vehicles used to dissolve the substances tested showed no significant action in the behavioral tests (data not shown).

### Models used for the evaluation of antinociceptive activity

#### Hot plate test

The hot plate temperature was kept at 54 ± 1°C. The response was considered in the form of jumping or licking of the paws. The latency (in seconds) was recorded before (from two consecutive measurements at intervals of 30 minutes) and after 90 min following the oral administration of BM-21 or indomethacin. This time was selected on the basis of preliminary experiments using 15 mg kg^-1 ^indomethacin showing that at this time the response reached a maximum, and then tends to decay. A cut-off time of 20 s was imposed to avoid damage to the paw. Analgesic activity was expressed as the increase in response time with respect to the corresponding pretreatment control.

#### Intraperitoneal acetic acid injection "writhing test"

The BM-21 extract, indomethacin, or water were administered 90 min before the test. After 85 min of BM-21 or indomethacin administration, mice were injected intraperitoneally (ip) with an aqueous solution of acetic acid (0.6%). The number of contortions and stretches in each mouse was counted for 20 minutes, starting 5 min after acetic acid injection. The results were expressed as percentage of change with respect to controls.

#### Formalin test

Mice were habituated for 30 min in the glass observation chambers (20 × 20 × 30 cm). After 90 min, BM-21, thalassiolin B, amiloride, or indomethacin were administered. All mice received 20 μL of 5% formalin injected into the plantar surface of the right hind paw. The total number of licking-biting of the injected paw during the acute early phase (0-5 minutes) and the tonic, last phase (15-30 minutes) after formalin injection were measured.

#### Evaluation of motor activity

The motor activity of the animals was evaluated in a rota-rod apparatus. The animals were placed on the rota-rod (16 and 32 rpm) and the time they remained on the apparatus was recorded. The cut off time was 2 min. The performance time was measured for control animals and at 40, 400, and 1000 mg kg^-1 ^BM-21. The rota-rod test was made 90 min after BM-21 administration.

#### Statistical analysis

ANOVA tests and the nonparametric analysis of variance (Kruskal-Wallis, and U Mann-Whitney) tests were used to evaluate statistical significance. The software used was SPSS (IBM) and differences between treated and control groups were considered statistically significant when *P *≤ 0.05.

### Electrophysiological experiments in vitro

To study the effect of BM-21 and of thalassiolin B on the ASIC current, the whole cell patch-clamp technique was used. For this purpose the DRG neurons from Wistar rats (P5 to P9) were isolated and cultured according to the procedure described by Salceda and coworkers [19].

#### Cell culture

The rats were anesthetized and killed with an overdose of sevofluorane. The dorsal root ganglia were isolated from the vertebral column and incubated (30 min at 37 °C) in Leibovitz L15 medium (L15) (Invitrogen, Carlsbad, CA) containing 1.25 mg mL^-1 ^trypsin and 1.25 mg mL^-1 ^collagenase (both from Sigma-Aldrich, St. Louis, MO, USA). After the enzyme treatment, the ganglia were washed 3 times with sterile L15. Cells were mechanically dissociated using a Pasteur pipette and then plated on 12-mm × 10-mm glass coverslips (Corning, Corning, NY) pretreated with poly-D-lysine (Sigma-Aldrich) and placed onto 35-mm culture dishes (Corning). Neurons were incubated 4 to 8 h in a humidified atmosphere (95% air, 5% CO_2_, at 37 °C) using a CO_2 _water-jacketed incubator (Nuaire, Plymouth, MN) to allow the isolated cells to settle and adhere to the coverslips. The plating medium contained L15, with added 15.7 mM NaHCO_3 _(Merck, Naucalpan, Mexico), 10% fetal bovine serum, 2.5 μg/mL fungizone (both from Invitrogen), 100 U/mL penicillin (Lakeside, Toluca, Mexico), and 15.8 mM HEPES (Sigma-Aldrich).

#### Electrophysiological recording

The DRG neurons were incubated 4 to 8 h and the culture dish with attached cells was mounted on the stage of an inverted phase-contrast microscope (TMS, Nikon Co. Tokyo, Japan). Cells were bathed in an external solution shown in Table 1. Cell voltage responses were studied at room temperature (23-25 °C) using an Axopatch 1D amplifier (Molecular Devices, Union City, CA). The cells for recording were selected as not adhered to other cells, to show no neurite outgrowth, and to have a round soma of regular size. Only cells showing a distinct peak-response (an initial peak followed by sustained current) to acid perfusion were used for recording. Command-pulse generation and data sampling were controlled by pClamp 8.0 software (Molecular Devices) using a 16-bit data-acquisition system (Digidata 1320, Molecular Devices). Signals were low-pass filtered at 5 kHz and digitized at 10 kHz. Patch pipettes were pulled from borosilicate glass capillaries (TW120-3; WPI, Sarasota, FL) using a Flaming-Brown electrode puller (80-PC; Sutter Instruments, San Rafael, CA). They typically had a resistance of 1 to 3 MΩ when filled with the intracellular solution (see Table 1). The series resistance was electronically compensated for by ≈ 80%. The digital data were stored in a PC computer for off-line analysis.

**Table 1:** Solutions used for voltage clamp recording.

	Extracellular (mM)	pH 6.1 Extracellular (mM)	Intracellular (mM)
**NaCl**	140	140	10

**KCl**	5.4	5.4	140

**CaCl**_**2**_	1.8	1.8	0.134

**MgCl**_**2**_	1.2	1.2	-

**HEPES**	10	-	5

**MES**	-	10	-

**EGTA**	-	-	10

**MgATP**	-	-	2

**Na-GTP**	-	-	1

	pH 7.4 adjusted with NaOH	pH 6.1 adjusted with NaOH	pH 7.2 adjusted with KOH

#### Experimental protocols and data analysis

The ASIC currents were generated by a fast (about 100 ms) pH change from 7.4 to 6.1 for 5 seconds, by shifting one of the three outlets of a fast change perfusion system (SF-77B, Warner Inst., Hamden, CT) while keeping the cell at a holding potential (V_h_) of -60 mV. The time between the pH step changes was 1 minute to guarantee that the ASIC current was completely recovered from desensitization. The transient receptor potential V1 (TRPV1) antagonist capsazepine (10 μM) (Sigma Chemical Co., St Louis MO) was added to the extracellular solution (pH 6.1) to avoid potential activation of the TRPV1 receptor by a drop in pH. The variables measured to characterize the ASIC currents were a) maximum peak amplitude, b) current desensitization time-constant (determined by fitting the decay phase of the current with a single exponential function: τ_des_), c) the amplitude at the end of the acid pH pulse (it was considered a measure of the steady-state current and normalized in relation to peak current). The BM-21 was coapplied with the pH change to 6.1 whereas the thalassiolin B was preapplied 20 seconds before the pH change to 6.1 and during the whole pH pulse [20]. The reason for this difference in application protocol was because thalassiolin B produced highly variable results when coapplied but a consistent effect when it was preapplied. The pH of the perfusion solution was checked after adding BM-21 or thalassiolin B so we can discard the possibility of pH changes caused by them. To characterize the DRG acid-gated currents, amiloride (10, 30, and 100 μM), Gd^3+ ^(100 μM), Na sulfate, and choline chloride were used.

The concentration-response relationship was obtained comparing the effect of BM21 and of thalassiolin B with its control. The data were fitted with the function *Y *= *min *+ (*max*-*min*)/(1+(*x*/*EC*_*50*_)^H^), where *Y *is the pharmacological effect of the substance under study, *x *is the substance concentration, *max *and *min *are the maximum and the minimum effects, *EC*_*50 *_is the concentration at which 50% of the effect is obtained and H is the Hill slope constant. To define the statistical significance a paired Student's *t*-test was used and *P *≤ 0.05 was considered as significant. Experimental data are presented as the mean ± standard error.

## Results

### Concentration of thalassiolin B in the crude extract of *Thalassia testudinum*

**Figure 1:**
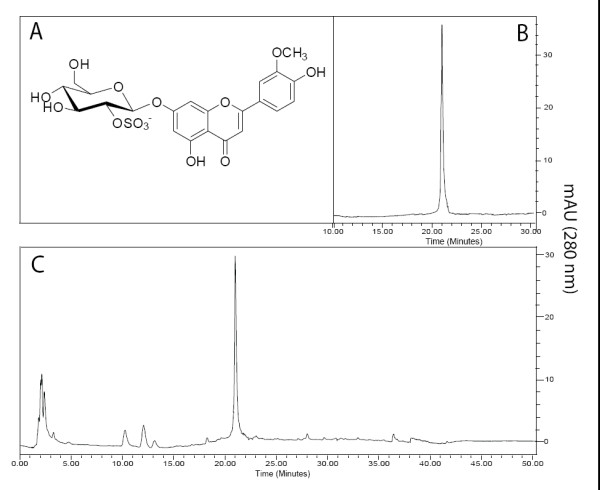
**Chromatographic profile of BM-21 and structure of the bioactive flavone glycoside**. A) Chemical structure of thalassiolin B (chrysoeriol 7-β-D-glucopyranosyl-2"-sulphate) isolated from *T. testudinum*. B) HPLC traces of the standard of thalassiolin B. C) HPLC profile of BM-21. The HPLC conditions to obtain B and C were similar (see 'Material and Methods').

The concentration of thalassiolin B in the crude extract BM-21 obtained from *Thalassia testudinum *was determined. Because thalassiolin B is water soluble and difficult to isolate quantitatively, it was necessary to evaluate its concentration by using analytical-scale HPLC peak integration. Using this method, the concentration of thalassiolin B was equivalent to 5.8 ± 0.3% (weight) of the crude extract. The HPLC traces of this compound (standard) and of the crude extract are shown in Figure 1. The absorbance spectra for the standard and the corresponding 21-min peak in the extract were identical, confirming that the compound being quantified was thalassiolin B. This was also confirmed by LC-ESIMS analysis which showed the characteristic ion at *m/z *541 ([M-H]^-^).

### Effects of BM-21 and thalassiolin B in vivo

#### Hot plate test

**Figure 2:**
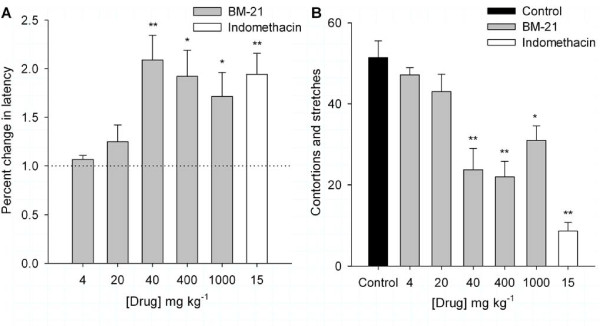
**Effects of BM-21 on the thermal nociception and acetic acid writhing test**. A) In the hot plate test the use of BM-21 (40 - 1000 mg kg^-1^) and 15 mg kg^-1 ^indomethacin increased the latency of response measured 90 min after drug administration. Control = 3.3 s (dotted line). * *P *≤ 0.05, ** *P *≤ 0.001. In this and following graphs each bar represents mean ± SE, marks * and ** indicate significantly different from control (*P *≤ 0.05, *P *≤ 0.001) (one-way ANOVA and Student's *t*-tests). B) Number of contortions and stretches generated by the intraperitoneal injection of acetic acid measured in control and after 90 min of BM-21 or indomethacin administration.

The BM-21 was used in concentrations ranging from 4 to 1000 mg kg^-1 ^in the diverse models of nociception. In the hot plate test, the administration of 4 and 20 mg kg^-1 ^BM-21 (*n *= 6 each) produced no significant effect. However BM-21 (40, 100, 400, and 1000 mg kg^-1^; *n *= 12 each) and indomethacin (15 mg kg^-1^; *n *= 16) produced a significant increase in the response latency tested 90 min after its administration (*P *≤ 0.05; Figure 2A), although the action of 400 and 1000 mg.kg^-1 ^BM-21 had a lower amplitude than that produced with 40 mg kg^-1^.

#### Writhing test

The ip injection of acetic acid in control mice produced 51.4 ± 4.1 contortions and stretches (*n *= 19). The action of BM-21 and indomethacin were studied 90 min after their administration. The use of 4 and 20 mg kg^-1 ^BM-21 (*n *= 8 each) apparently reduced the number of contortions and stretches but this reduction was not significant (*P *≥ 0.05). The use of a higher concentration of BM-21 (40 mg kg^-1^) significantly reduced the number of contortions and stretches to 26.8 ± 5.7 (47% protection, *n *= 12; *P *= 0.018), and 400 mg kg^-1 ^BM-21 produced the maximum reduction in the number of contortions and stretches; 21.9 ± 3.9 (57% protection; *n *= 19; *P *≤ 0.001), whereas at 1000 mg kg^-1 ^the reduction was 31 ± 3.5 (39% protection; *n *= 10; *P *= 0.004) (Figure 2B). In the experimental group in which 15 mg kg^-1 ^indomethacin was used (*n *= 12) the number of contortions and stretches was 8.7 ± 2.1 (83% protection; *P *≤ 0.001 compared to control). The action of indomethacin was apparently greater than the maximum effect of 400 mg kg^-1 ^BM-21 but the difference was not significant (*P *= 0.19).

Because the maximum reduction of contortions and stretches was obtained with 400 mg kg^-1 ^BM-21, this dose was used to study the temporal dependence of the BM 21 effect in the acetic acid-generated contortions and stretches. The effect of 400 mg kg^-1 ^BM-21 was significant after 60 minutes (34% reduction; *P *= 0.03) and a greater reduction of contortions and stretches was reached after 90 minutes (57% reduction; *P *= 0.001) of BM-21 administration (data not shown).

#### Formalin test

**Figure 3:**
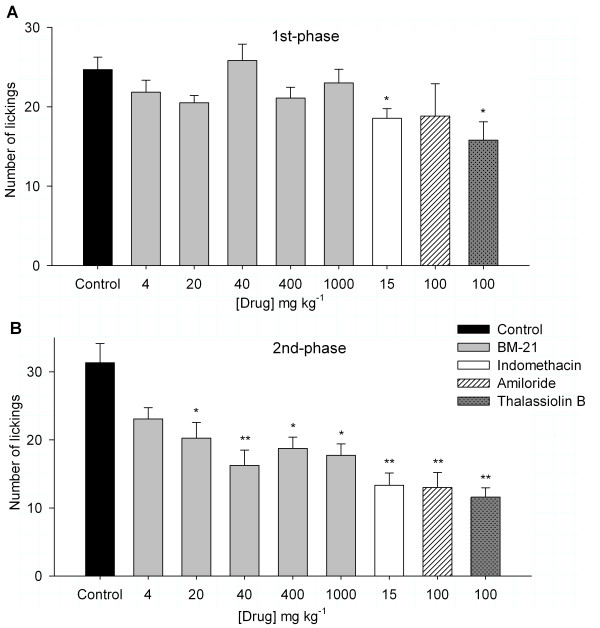
**The effect of BM-21 and thalassiolin B on nociception caused by formalin injection into the hind paw**. A) No significant effect of BM-21 or amiloride was observed during the 1st-phase nociception, although indomethacin and thalassiolin B produced a significant reduction in the number of lickings. B) In the 2nd-phase, except for the lowest dose of the BM-21 (4 mg kg^-1^), all the drugs used produced a significant reduction in the number of lickings. The magnitude of the thalassiolin B action was similar to that of amiloride and apparently larger than that produced by 15 mg kg^-1 ^indomethacin, but the difference was not significant (*P *= 0.302).

In the formalin-generated licking behavior the administration of 4 to1000 mg kg^-1 ^BM-21 (*n *= 12 for each group) had no significant effect on the number of lickings in the 1st-phase (0-5 min) after formalin injection (Figure 3A). However, in the 2nd phase (15-30 min), the BM-21 produced a significant effect at concentrations ranging from 20 to 1000 mg kg^-1 ^The maximum reduction of 49% in the licking behavior was attained with the use of 40 mg kg^-1 ^BM-21. In comparison with the control group, a one-way ANOVA analysis revealed a significant effect of the extract in the late phase at doses of BM-21 from 20 to 1000 mg kg^-1 ^(Figure 3B). The use of 15 mg kg^-1 ^indomethacin produced a significant reduction in both the 1st- and 2nd-phase of the formalin test. The licking behavior during the 1st-phase was unaffected by 100 mmol kg^-1 ^amiloride (*n *= 6), whereas 100 mmol kg^-1 ^thalassiolin B (*n *= 6) decreased the licking behavior 34% (Figure 3A). Both compounds reduced the 2nd-phase licking behavior similarly (13 ± 2.2 for amiloride and 11.6 ± 1.4 for thalassiolin B; Figure 3B).

The actions of BM-21 in the nociception tests used did not show typical dose response relationships, which may have been caused by the nonspecific actions of BM-21. In the work with natural extracts it is common to find this type of dose relationship. This extract is a mixture of flavonoids, terpenes, saponins, and other constituents that could contribute differently to the biological activity. The antinocioceptive effect of BM-21 results from the combined action of all the pharmacologically active components of the extract. It would be possible that the pharmacological action of one or more compounds could produce opposite effects (pronociceptive action) and at higher concentrations this contribution could have become more evident in comparison with other compounds that can reach a plateau at this concentration, resulting in a decrease of the action of the BM-21. Considerable mechanistic evidence indicates that U-shaped dose-response curves are common and have been designated as hormetic effects, mediated by agonist concentration gradients with different affinities for stimulatory and inhibitory regulatory pathways [21].

#### Motor activity

To discard the possibility that the BM-21 would exert a sedative action, explaining the effect observed in previous behavioral tests, studies of the motor activity of the animals after BM-21 (40, 400, and 1000 mg kg^-1^) were made using the rota-rod test. According to the data shown in Table 2 (*n *= 12), BM-21 did not alter the time that mice spent in the rota-rod apparatus, indicating that potential changes in the motor activity could not account for the effects observed in previous behavioral tests, thus supporting that BM-21 action is caused by an antinociceptive effect.

**Table 2:** Rota-Rod motor performance under the influence of BM-21.

	Latency		
	8 rpm	16 rpm	32 rpm

Control	120	120	120

40 mg kg^-1^	120	117 ± 2.3	117 ± 1.8

400 mg kg^-1^	120	120 ± 0.1	117 ± 2.1

1000 mg kg^-1^	120	120	108 ± 7.2

Table shows the latency of the time that mice spent in the rota-rod apparatus in control and with the use of various BM-21 concentrations (cut-off time was 120 s).

### Effects of BM-21 and thalassiolin B in vitro

#### DRG acid-gated currents

**Figure 4:**
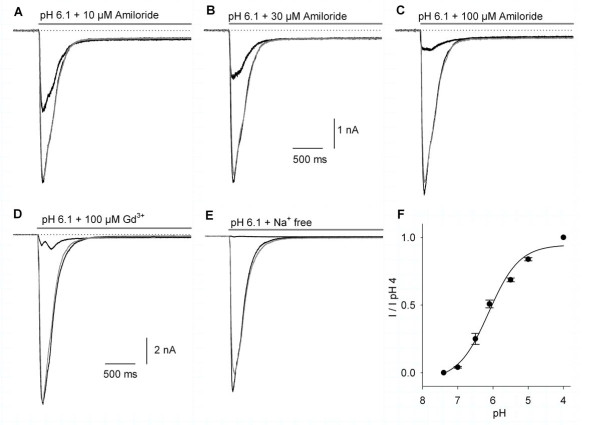
**The effect of amiloride, Gd**^**3+**^**, and Na**^**+**^**-free extracellular solution on the proton-gated current in DRG neurons**. A-C) The use of amiloride produced a concentration-dependent and reversible inhibition of the proton-gated peak current without significantly modifying the steady-state current. In this and following graphs the black traces represent the control and the drug effect, the gray trace is after washout. The dotted line shown in this and following current recordings indicates the zero current, and the gray bar the time of pH 6.1 + drug application. The graphs display the first 3 seconds after the 5-s pH 6.1 perfusion. D) The use of Gd^3+ ^also produced a reversible inhibition of the proton-gated current. E) The use of a Na^+^-free extracellular solution significantly decreased the current (to 4% of control current) reversibly. F) The normalized current amplitude versus pH relationship shows a sigmoidal dependence on the pH with a pH_50 _of 6.12 ± 0.1. Currents were normalized as a function of the current obtained with pH 4.

The whole-cell voltage-clamp recordings of acid-gated currents were obtained on 114 DRG neurons. The mean membrane-capacitance of the cells was 59 ± 1.3 pF, corresponding to an estimated neuron diameter of 43 ± 5 μm. The cells used for the recordings constitute a relatively restricted population primarily from large-diameter DRG neurons. The current activated by acidic-solution perfusion in DRG neurons showed characteristics indicating the participation of diverse ASIC subunits in the macroscopic current. The acidic-gated current characteristics found in DRG neurons varied from a fast transient current with little steady-state current, to a current with a clear steady-state, and finally to a slowly desensitizing current with a large steady-state component. To demonstrate that the acid-gated currents that were recorded from the DRG neurons in our experimental conditions were indeed caused by the activation of the ASICs, the use of 10, 30, and 100 μM amiloride produced a concentration dependent 39 ± 3.8% (*n *= 3), 67 ± 4.5% (*n *= 7), and 85 ± 2.3% (*n *= 6) reversible inhibition of the proton-gated peak current (Figure 4A, B, C). Also, the use of 100 μM Gd^3+ ^(*n *= 4) produced a significant inhibition of 90 ± 1.3% of the acid-gated current (Figures 4D). The acid-gated currents were also studied with the use of an external solution in which 140 mM NaCl was substituted by 140 mM choline chloride (*n *= 5). In this condition the inward current produced by a pH 6.1 acid solution perfusion was reduced 96 ± 1% compared to the control, showing that the proton-gated current was an Na^+ ^current (Figure 4E). The curve for the current amplitude versus pH showed that half of the maximum current (pH_50_) was attained at pH 6.12 ± 0.1 (*n *= 7; Figure 4F). These results demonstrate that the proton-gated current we are recording had the typical properties of the ASIC currents; a proton-gated Na^+ ^current inhibited by amiloride and Gd^3+ ^(both in the μM range).

**Figure 5:**
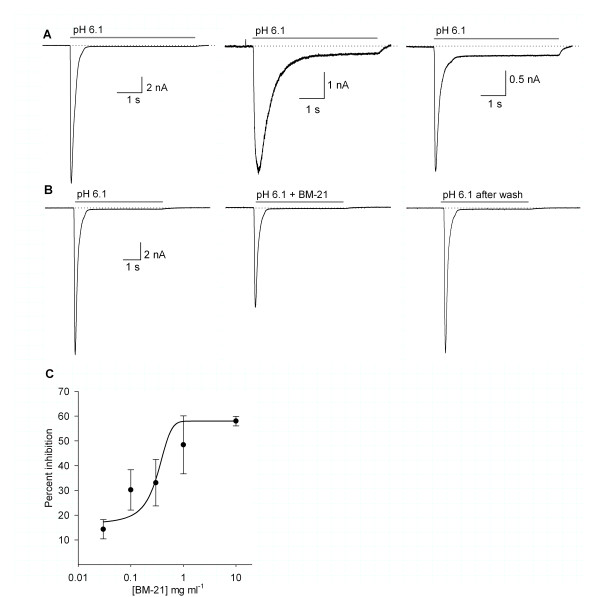
**ASIC-current type and effect of BM-21 on the ASIC currents**. A) Typical pH-gated currents recorded in DRG cells (pH 6.1, 5 s). B) The use of 10 mg mL^-1 ^BM-21 reduced the peak amplitude of the pH-gated current in a reversible form. C) The dose-response relationship of the BM-21 inhibitory effect on the ASIC currents in the DRG neurons was constructed pulling together cells with a desensitizing τ < 400 ms. The IC_50 _was 0.72 ± 0.16 mg mL^-1^.

Based on the pH sensitivity of the ASIC currents we decided to use a pH of 6.1 to activate the ASIC currents in the experiments designed to characterize the action of the BM-21 and thalassiolin B on the ASIC currents in the DRG neurons. It was possible to distinguish two main types of acid-gated currents; a fast transient current with a complete desensitization or with a small steady-state current (less than 10% of the peak current), and a slowly desensitizing current (τ greater than 400 ms). From their kinetics and desensitization profiles, it was possible to establish an analogy between the first type and the respective cloned ASIC1- and ASIC3-like and the second type as the ASIC2-like [9] (Figure 5A). It was not possible to find any significant correlation between the ASIC current characteristics and cell capacitance, perhaps because other authors used acutely dissociated instead of cultured cells [9]. The ASIC2-like current was clearly distinguishable and its incidence was low (about 25% in frequency). However, it was not possible to separate ASIC1- and ASIC3-like currents, because the proportion of different channel populations within the same cell generates a wide range of possible current waveforms. So, for practical purposes we classified the ASIC currents as fast (with a τ < 400 ms) and slowly desensitizing (with a τ > 400 ms).

None of the substances used in this work produced any electrophysiological action or any changes in the holding current by their perfusion alone. This excludes the possibility that their actions were caused by alterations of the many other ionic currents present in DRG neurons at -60 mV.

#### Effect of BM-21 on the ASIC currents

The coapplication of BM-21 with pH 6.1 reduced the peak amplitude of the rapid desensitizing ASIC currents in the doses assayed in a concentration-dependent manner. The BM-21 had no significant effect on the ASIC currents with a slowly desensitizing time-course (τ > 400 ms). At the highest concentration used (10 mg mL^-1^) the amplitude of the of rapid desensitizing (τ < 400 ms) ASIC current was reduced 56% from 8.1 ± 2.2 nA in the control to 3.7 ± 1.1 nA (Figure 5B). The concentration response relationship had an IC_50 _of 0.72 ± 0.16 mg mL^-1 ^(*n *= 4; Figure 5C).There was a no significant tendency to increase the amplitude of the sustained component by increasing the BM-21 concentration. The current desensitization rate was not significantly affected (control τ = 231.7 ± 23.3 ms versus τ = 319.6 ± 56.6 ms, *P *> 0.05 Student's *t *test, with 10 mg mL^-1 ^BM-21), and its action was fully reversible after washing the preparation (between the first and the second minute) at concentrations below 10 mg mL^-1^. However, the maximum current inhibition of 56% was attained with 10 mg mL^-1 ^BM-21 and the reversibility at this dose was 84%.

#### Effect of thalassiolin B on the ASIC currents

**Figure 6:**
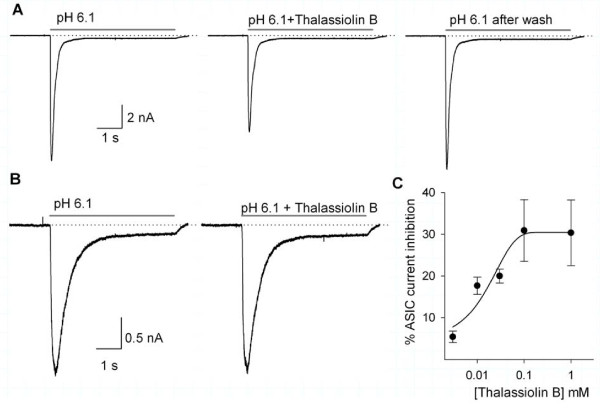
**The effect of thalassiolin B on the ASIC currents**. A) Inhibitory action of 0.03 mM thalassiolin B on the ASIC current in a cell with a τ = 283.3 ms was 23% and fully reversible. B) No significant action of 0.03 mM thalassiolin B was observed in slowly desensitizing ASIC currents. Current shown has a τ = 984 ms. C) Concentration-response relationship of thalassiolin B inhibitory action on the ASIC currents in DRG neurons. The continuous line shows a dose-response curve adjustment to the data with an IC_50 _of 27 ± 2.6 μM.

The use of thalassiolin B also produced a significant reduction of the ASIC-current peak amplitude in those cells with a fast desensitizing current (τ < 400 ms), with no significant change in the kinetic characteristics of the current (Figure 6A). No action was exerted by this compound on the slowly (τ > 400 ms) desensitizing ASIC currents (*n *= 8; Figure 6B). The maximum inhibitory action of thalassiolin B was 30%, from 5.6 ± 1.6 nA under control conditions to 3.9 ± 1.0 nA in the presence of 1 mM thalassiolin B. The concentration-effect relationship for the thalassiolin B-sensitive currents showed a dose-dependent inhibitory action on the peak-current amplitude with an IC_50 _of 27 ± 2.6 μM (*n *= 5; Figure 6C). The effect was fully reversible at all the concentrations used and the maximum current inhibition of about 30% was attained with 0.1 mM thalassiolin B. The current desensitization time-constant was not significantly affected (control τ = 207 ± 11 ms versus τ = 192 ± 14 ms, *P *> 0.05, Student's *t *test, with 1 mM thalassiolin B). Also, the sustained component of the ASIC current was unaffected.

Because thalassiolin B is a sulfate flavone glycoside, for a control of the potential effect of sulfate the perfusion of 0.1 mM sodium sulfate was also studied. No significant effect of sodium sulfate on the ASIC current was found (*n *= 4; data not shown), indicating that the observed effects were specifically caused by the molecule of thalassiolin B.

**Figure 7:**
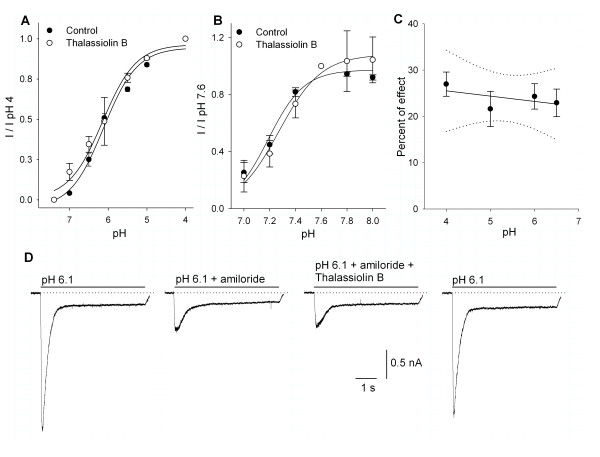
**The effect of thalassiolin B on the pH versus amplitude and steady state desensitization curve of the ASIC current**. A) No significant influence of 100 μM thalassiolin B on the pH dependence of the ASIC current amplitude was found. B) The use of thalassiolin B also did not significantly modify the steady-state desensitization of ASIC currents. C) Analysis of the thalassiolin B effect as function of pH used to activate the current shows no significant correlation (*P *> 0.05). Line shows the linear regression (slope = 0.27); dotted lines show confidence intervals at *P *≤ 0.05. D) Amiloride (30 μM) decreased the current 64 ± 4%. The coapplication of 30 μM amiloride and 100 μM thalassiolin B produced an additional nonsignificant 10.8 ± 3.8% decrease of the current (*P *≥ 0.05 Student's *t*-test; thalassiolin B preapplied 20 s before coapplication of amiloride and thalassiolin B). Effects were fully reversible.

To study the influence of thalassiolin B on the gating of the ASIC current, the pH dependence of the current amplitude was studied in the control and with the use of 100 μM thalassiolin B. The pH50 in the control was 6.12 ± 0.1 (*n *= 7) and with the use of thalassiolin B was 6.15 ± 0.1 (*n *= 7; *P *≥ 0.05; Figure 7A). This result indicates no interaction with the proton gating of the channel. To further characterize the potential influence of the occupation of proton-binding sites on the actions of thalassiolin B, the effects of the drug were studied while the pH of the bath solution was varied between 7.0 and 8.0 with 0.2 pH increments (steady-state desensitization curves). The pH of the preconditioning bath produced a significant effect over the current produced by perfusion at pH 6.1. The preconditioning pH-effect relationships were constructed in control (pH_50 _= 7.19 ± 0.04, *n *= 5) and in the presence of 30 μM thalassiolin B (pH_50 _= 7.27 ± 0.03, *n *= 7; Figure 7B), indicating that its action is not dependent on the state, closed or inactivated, of the ASICs. The relationship between the effect of 100 μM thalassiolin B and the pH used to activate the ASIC current showed no significant pH dependence (pH 4 *n *= 22; pH 5 *n *= 8; pH 6.1 *n *= 8; pH 6.5 *n *= 9; Figure 7C). The interaction of amiloride and thalassiolin B was also studied. We found that the effect of 100 μM thalassiolin B on the ASIC current activated by pH 6.1 was occluded by the use of 30 μM amiloride (*n *= 5; Figure 7D). Finally, no significant influence of holding voltage (-20 mV versus -60 mV) on the thalassiolin B action was observed (*n *= 4; data not shown).

## Discussion

This study demonstrates the antinociceptive action of BM-21 in the classical pharmacological models of thermal- (hot plate test) and chemical-caused (acetic acid and formalin tests) pain in mice and of thalassiolin B in the formalin test. The inhibition of the nociceptive behavior does not seem to result from a nonspecific muscle-relaxant or sedative effect because the BM-21 extract did not show any motor performance alterations in the rota-rod test.

The hot plate test is one of the most commonly used for nociception studies [22]. The increase in temperature produces two behavioral components (paw licking and jumping) that can be measured by their reaction times. In this model, BM-21 significantly increased the latency for jumping or licking suggesting that the extract exerts an acute antinociceptive action [23]. The acetic acid-generated writhing test has been used for the assessment of analgesic or antiinflammatory properties of several drugs. Oral administration of BM-21 reduced contortions and stretches thus indicating it is exerting an antinociceptive action in persistent tonic pain.

Nociceptive behavior in the formalin test has been divided into two phases [22,24]. The initial 1st-phase (nociceptive pain) is considered to be caused predominantly by activation of C fibers, whereas the 2nd-phase is associated with inflammatory components with release of different pain mediators (22,23,24). Reactive oxygen species (ROS) have been documented to contribute to and maintain conditions of chronic pain [25]. Both phases are sensitive to centrally acting drugs such as opioids [26], but the 2nd-phase is also sensitive to corticosteroids and nonsteroidal antiinflammatory drugs (NSAIDs), especially when the formalin is injected in high concentrations [26-29]. Antioxidants attenuate both phases of the responses in mice [25]. It has been established that COX-1 is involved in the 2nd-phase response in the formalin test and in the writhing test in mice [30]. A previous report has demonstrated the inhibition in vitro of the activity of both phospholipase A2 and COX-1 by BM-21[31]. Thus, the dual effect exerted by BM-21 on the arachidonic acid pathway could account for its effect in the 2nd-phase of the formalin test and in the writhing test. The BM-21 and thalassiolin B also have ROS scavenging properties. These could contribute to the antinociceptive effects observed in vivo, however in the DRG experiments in vitro this effect could be minor or absent. The contribution of ASIC channels in the formalin test has been documented and consistently both amiloride and thalassiolin B attenuated painful behavior in the late phase. For amiloride our data show similarities with those reported in the literature [32]. Albeit it has been found that amiloride use in the formalin test attenuated the 2nd-phase in female, but not in male, CD 1 mice (except at a toxic dose) [33]. The effect of BM-21 on both the formalin and writhing tests support that its profile is consistent with antiinflammatory analgesic drugs, however a phasic-antinociceptive action is also produced, particularly bearing in mind the antinociceptive effects of BM-21 on the hot plate model and the effect of thalassiolin B on the first phase of the formalin test.

Regalado and coworkers determined that the phenolic content in BM-21 was 18 ± 1.5% [17]. Consistent with this, the separation procedure of the plant extract resulted in the isolation of thalassiolin B as the major phenolic constituent of the extract. Phenolic compounds, and particularly flavonoids, have a wide variety of biological activities in mammals [34,35]. Our results indicate that flavonoids, in particular thalassiolin B, may contribute in a significant degree to the biological action of BM-21. In fact, the studies in vivo using the formalin test in mice support that thalassiolin B per se has an antinociceptive action.

Some mechanisms of nociception are known to involve ASICs [2]. Previous results have documented that they sense extracellular acidifications occurring during inflammation and their expression is increased in rat sensory neurons (particularly ASIC3) in this pathological condition [10]. Thus, in an attempt to characterize the mechanism through which the BM-21 exerts its antinociceptive action we evaluated BM-21 and thalassiolin B on ASIC currents.

We found that both BM-21 and thalassiolin B were able to decrease the peak amplitude of ASIC currents; indicating that, most probably, the effect of BM-21 on ASIC currents may be because of the presence of thalassiolin B. The maximum effect of BM-21 and thalassiolin B on ASICs currents was about 56% and 30%. Therefore the activity of thalassiolin B could not account for the overall activity of the extract, which suggests that other components are also able to act on the ASIC currents. The presence of other molecules in the extract and in particular of thalassiolins A and C could contribute to this synergic effect [18].

The thalassiolin B inhibitory action on ASIC currents in DRG neurons was selective, acting only in those neurons with a fast desensitization time-course, without significant effect on those currents with a slow desensitizing time-course, the so-called "ASIC2-like" in DRG neurons [9], indicating that its effect could be specific. However, because H^+^-gated currents in DRG neurons are likely to be caused by the combination of two or more ASIC subunits coassembled as heteromultimers, with a coexistence of multiple channel populations within the same cell [36,37], it is not possible to ascertain its selectivity.

Thalassiolin B acts in the same range of potency in DRG neurons (IC_50 _= 27.3 ± 2.6 μM) as A-317567 and amiloride. Both produce a concentration-dependent inhibition of ASIC currents with an IC_50 _ranging between 2 and 29 μM and 30 and 51 μM in acutely dissociated DRG neurons [9]. Their inhibitory action is similar to that of streptomycin (32 ± 2.7 μM), neomycin (44 ± 2.6 μM) [20], amiloride (30 to 51 μM), and A-317567 (2 to 29 μM) [9]. In contrast, APETx-2 (from the sea anemone *Anthopleura elegantissima*) in primary-cultured sensory neurons inhibits ASIC3-like currents with an IC_50 _of 216 nM [38], whereas psalmotoxin 1 (PcTx1, from the tarantula *Psalmopoeus cambridgei*) seems to be the most potent and inhibits a subpopulation of H^+^-gated currents (ASIC1a-like) in DRG neurons with an IC_50 _= 0.7 nM [39]. Similar to PcTx1 and APETx2 and in contrast with low-molecular-weight compounds of nonpeptidic nature, amiloride and A-317467 [9], thalassiolin B shows an effect that depends on the application schedule, and it needs to be applied a few seconds before the pH change, suggesting a complex interaction with the ASICs.

For its mechanism of action, thalassiolin B has a selective action among the different ASIC channel subunits sparing those currents with a τ ≥ 400 ms. The pH current curve shows that thalassiolin B did not modify the proton gating of the ASICs in DRG neurons. The steady-state desensitization curves also indicate that its action is not modified by channel desensitization. The effect of thalassiolin B shows no dependence on the pH used to activate the ASIC current, thus indicating that no conformational changes of the molecule as a function of pH are taking place. Interestingly, the coapplication of amiloride occluded the effect of thalassiolin B thus suggesting that they act through a similar mechanism or that the action of amiloride is downstream from thalassiolin B. Our data shows no voltage dependence of its effect indicating that most probably it is not entering into the membrane field potential. It requires preapplication to have a consistent inhibitory effect and it is able only to partially block the ASIC currents (about 30% at its highest concentrations), thus suggesting that thalassiolin B is not an ASIC-pore blocker. Future studies using heterologous expression of ASIC subunits would contribute to define the selectivity and action mechanism of thalassiolin B on the ASICs.

## Conclusions

The results of this study demonstrate the antinociceptive action of the *T. testudinum *extract, BM-21, after a single oral administration in classical thermal- and chemical caused pain in mice. The antinociceptive effects of BM-21 may be partially caused by its antagonism to ASIC channels. Thalassiolin B, the major phenolic constituent isolated from BM-21 also had an antinociceptive behavior in the formalin test and inhibited ASIC currents in DRG neurons indicating that this sulphated flavone glycoside may be responsible, at least in part, for the effects found with BM-21.

To our knowledge, this is the first report of the presence of an ASIC inhibitor in a marine-plant extract and associated to an identified phenolic compound. In comparison with other natural substances of peptidic nature with antinociceptive action such as PcTx1 and APETx2, the nonpeptidic nature of thalassiolin B may have an advantage for future therapeutic applications in the control of pain. However, further studies are still required to ascertain its selectivity within ASIC subunits, and to determine its potency, the time required to attain therapeutic levels in vivo, and its mechanism of action.

## Abbreviations

**APETx-2**: peptide from the sea anemone *Anthopleura elegantissima; ***ASIC**: acid sensing ionic channels; **COX-1**: cyclooxigenase-1; **DRG**: dorsal root ganglion; **EGTA**: ethylene glycol tetraacetic acid; **ESI**: electrospray ionization; **HEPES**: 4-(2 hydroxyethyl)-1-piperazineethanesulfonic acid; **HPLC**: high performance liquid chromatography; **IP**: intraperitoneal; **LC**: liquid chromatography; **MES**: 2-(N morpholino)ethanesulfonic acid; **MS**: mass spectrometry; **NMR**: nuclear magnetic resonance; **NO**: nitric oxide; **NSAID**: nonsteroidal antiinflammatory drugs; **PcTx1**: psalmotoxin 1 from the tarantula *Psalmopoeus cambridgei; ***ROS**: reactive oxygen species.

## Competing interests

The authors declare that they have no competing interests.

## Authors' contributions

AG, RM, TG, RAM made the behavioral studies on mice. AG, ES, OL and ESoto made the voltage clamp experiments including processing of the data. AL, OPT, ELR did the isolation and characterization of BM-21 and of thalassiolin B. AG and Esoto coordinated and helped with the writing of manuscript. All authors read and approved the final manuscript.
